# Relevance of comorbidities and antithrombotic medication as risk factors for reoperation in patients with chronic subdural hematoma

**DOI:** 10.1007/s10143-021-01537-x

**Published:** 2021-07-09

**Authors:** Alexander Younsi, Lennart Riemann, Cleo Habel, Jessica Fischer, Christopher Beynon, Andreas W. Unterberg, Klaus Zweckberger

**Affiliations:** Department of Neurosurgery, Heidelberg University Hospital, INF 400, 69120 Heidelberg, Germany

**Keywords:** Chronic subdural hematoma, Antithrombotics, Anticoagulants, Antiplatelets, Comorbidities, Reoperation, Risk factors

## Abstract

In an aging Western society, the incidence of chronic subdural hematomas (cSDH) is continuously increasing. In this study, we reviewed our clinical management of cSDH patients and identified predictive factors for the need of reoperation due to residual or recurrent hematomas with a focus on the use of antithrombotic drugs. In total, 623 patients who were treated for cSDH with surgical evacuation between 2006 and 2016 at our department were retrospectively analyzed. Clinical and radiological characteristics and laboratory parameters were investigated as possible predictors of reoperation with univariate and multivariate analyses. Additionally, clinical outcome measures were compared between patients on anticoagulants, on antiplatelets, and without antithrombotic medication. In univariate analyses, patients on anticoagulants and antiplatelets presented significantly more often with comorbidities, were significantly older, and their risk for perioperative complications was significantly increased. Nevertheless, their clinical outcome was comparable to that of patients without antithrombotics. In multivariate analysis, only the presence of comorbidities, but not antithrombotics, was an independent predictor for the need for reoperations. Patients on antithrombotics do not seem to necessarily have a significantly increased risk for residual hematomas or rebleeding requiring reoperation after cSDH evacuation. More precisely, the presence of predisposing comorbidities might be a key independent risk factor for reoperation. Importantly, the clinical outcomes after surgical evacuation of cSDH are comparable between patients on anticoagulants, antiplatelets, and without antithrombotics.

**Supplementary Information:**

The online version contains supplementary material available at 10.1007/s10143-021-01537-x.

## Introduction

Chronic subdural hematomas (cSDH) are among the most common neurosurgical conditions that predominately affect elderly patients [14]. Although commonly mild in its early symptoms, cSDH might turn into a severe disease with high recurrence and complication rates reaching 29%, finally resulting in increased mortality [32, 44]. In an aging society of industrialized countries, the incidence of this disease is expected to double in the next 20 years, becoming the most common neurosurgical condition by the year 2030 [6, 14]. This is accompanied by a rising complexity of patients, commonly presenting with underlying chronic diseases and being treated with antithrombotic medication [17].

Despite these upcoming challenges, several aspects of the clinical management of cSDH, predictors for its recurrence, and the role of antithrombotics on recurrence rates and clinical outcome are still unclear. In recent decades, numerous predictors for recurrence have been suggested, including epidemiological characteristics, radiological findings, clinical symptoms, and even laboratory values: Examples of such prognostic factors are higher age, male sex, hypertension, anticoagulants, hyperdense hematoma components or midline shift on preoperative imaging, and also elevated blood urea nitrogen or low levels of high-density lipoprotein. However, the results between various studies have remained conflicting and partly contradictory [13, 26, 31, 33, 34, 44, 46]. Especially the effects of antithrombotics on the risk of re-hemorrhage and the clinical outcome are widely disputed. Several studies showed an increased risk for cSDH recurrence in patients on antithrombotics, including a recent meta-analysis in which anticoagulants, as well as antiplatelets, were identified as risk factors [13, 33, 47]. Other authors, however, could not find such associations, and in another recent meta-analysis, antiplatelets but not anticoagulants were linked to postoperative cSDH recurrence [3, 15, 34, 38].

In this study, we sought to analyze the characteristics and management of surgically treated cSDH patients on anticoagulants and antiplatelets with respect to clinical outcome and complications. Furthermore, we aimed to identify factors predicting the need for reoperation due to residual hematomas or rebleeding.

## Materials and methods

### Patients

**Fig. 1:**
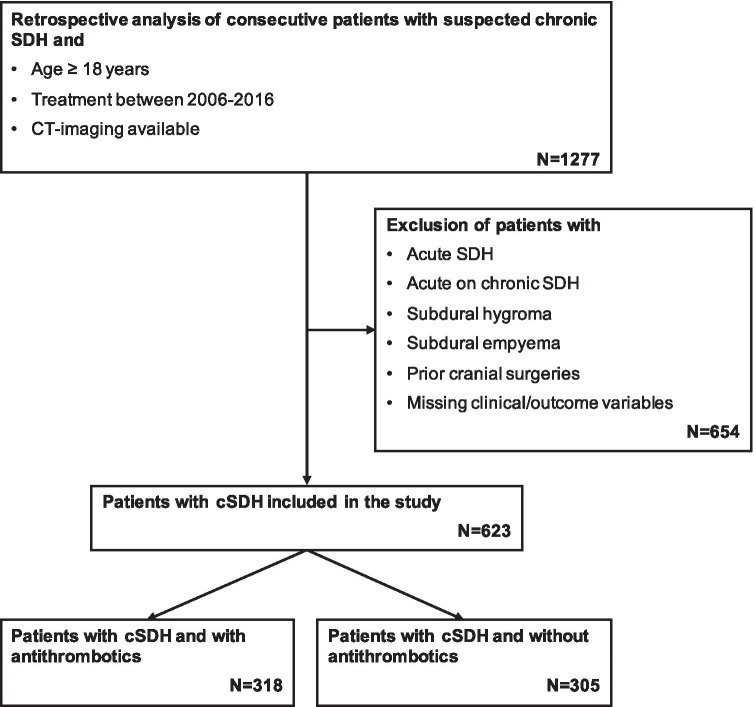
Flow diagram of patient selection

The medical charts of consecutive adults (18 years or older) treated surgically for cSDH at a single neurosurgical department from 2006 to 2016 were retrospectively analyzed. All cases of acute SDH, acute on chronic SDH, subdural hygroma, or subdural empyema, as well as all patients with prior cranial surgeries, were excluded (Fig. 1). All methods were carried out following relevant guidelines and regulations (Declaration of Helsinki). The standing committee of ethical practice of the Medical Faculty of the University of Heidelberg, Heidelberg, Germany, approved this study’s protocol. It waived the consent from analyzed patients due to its retrospective design.

### Treatment characteristics

Patients with cSDH confirmed by head CT were prepared for surgical evacuation if presenting with focal symptoms, deterioration in neurological status, or clinical signs such as headaches, combined with relevant radiologic hematoma appearance. The decision to operate was taken by the consultant neurosurgeon in charge. Patients were subjected to a blood panel that included the coagulation assays, international normalized ratio (INR), activated partial thromboplastin time (aPTT), and a platelet count, and in some cases, the assessment of platelet function. Although there was no unified institutional protocol for anticoagulation reversal, INR values < 1.3 were generally pursued in patients on antithrombotics for adequate hemostasis. In the case of patients on vitamin K antagonists (VKA), prothrombin complex concentrate (PCC) therapy was considered the first-line treatment, and two different 4-factor PCC products were used depending on their availability (Beriplex P/N® (CSL Behring, Marburg, Germany) and Octaplex® (Octapharma, Langenfeld, Germany)). As a general practice, administration of antithrombotic drugs was stopped on the day of admission if not urgently required due to, e.g., new coronary vessel stents and was resumed 14 days after surgery.

Patients with cSDH were usually treated with burr hole trepanation under intubation anesthesia. If present, inner membranes and/or septations were opened intraoperatively, and subsequent subdural drainage was performed for 48 h in supine position postoperatively as a standard practice. Of note, steroids were not peri-operatively administered to any patient. Thromboprophylaxis was carried out using elastic stockings and subcutaneous administration of low molecular weight heparin (Enoxaparin, Clexane®, Sanofi-Aventis, Frankfurt, Germany) once daily (usually 4000 I.U.), starting within 24 h after surgery. CT scans after hematoma evacuation were only performed when the neurological status deteriorated or when preoperative symptoms did not improve. The decision for reoperation was based primarily on clinical findings in combination with CT imaging (e.g., continued or increased midline shift). The second evacuation surgery was performed with a repeat burr hole drainage or a craniotomy, depending on the residual or recurrent hematoma’s extent and consistency.

### Outcome variables and end points

The following patient and treatment characteristics were collected: sex, age, cSDH etiology, laboratory values, radiological features on CT imaging, initial Glasgow Coma Scale (GCS), modified Rankin Scale (mRS), first symptom, main symptom, comorbidities, antithrombotics during the last 7 days before admission, surgical procedure, complications (cardiovascular, pulmonary, coagulation, or neurological), clinical course, in-hospital mortality, Glasgow Outcome Scale (GOS), and mRS on discharge. The variable “history of chronic comorbidity” refers to patients having at least one comorbidity (yes/no). The exact duration of the pausing of antithrombotic medication prior to and after the primary operation was not taken into account.

This study’s primary endpoint was the risk of reoperation (due to residual or recurrent hematomas) within 30 days after the first surgical intervention. Secondary endpoints were clinical outcome measures on discharge.

### Statistical analysis

Categorical variables were tested with the chi-squared test followed by pairwise post hoc tests with *p*-value adjustment for multiple testing. The Mann–Whitney *U* test was used for continuous variables between two and the Kruskal–Wallis test followed by a post hoc Dunn’s test between multiple groups. For the comparative analyses, patients were excluded when being on both anticoagulant and antiplatelet medication. Univariate and multivariate logistic regression regarding reoperation within 30 days with the most plausibly important variables age, gender, comorbidity, antithrombotic medication, and cSDH thickness as predictors was performed. Statistical significance was defined by *p*-values < 0.05. Results are given, unless stated otherwise, as median ± interquartile range (IQR). All statistical analyses were conducted using the software R (Version 3.5.1) [40].

## Results

### Patient and treatment demographics

A total of 623 patients (median age: 75 [68–81] years, male/female ratio: 2.24) who underwent surgical cSDH evacuation during the study period were included (Fig. 1). Headaches (28%), coordination deficits (24%), aphasia (11%), and confusion (11%) were the most common initial symptoms that patients reported. The most common leading symptoms at admission were coordination deficits (26%), hemiparesis (15%), aphasia (14%), and reduced vigilance (14%). The prevalence of comorbidities in the cSDH patient population was high (63%), and arterial hypertension, as well as cardiac arrhythmias, accounted for most of them (35% and 21%, respectively). On head CT imaging, cSDH had a median thickness of 21 [15-25] mm, were right-sided in 31%, left-sided in 41%, and bilateral in 28% of cases, while a midline shift was visible in 67% with a median of 6 [2-10] mm. Antithrombotics were present in more than half of all cSDH patients (51%) and were almost equally distributed between only anticoagulant (47%) and only antiplatelet (50%) drugs, with few patients (3%) receiving both. Substances used for anticoagulation were either VKA (89%), new oral anticoagulants (10%), or heparin (1%). Inhibition of platelet function was mostly induced with acetylsalicylic acid (88%) or with clopidogrel (3%) and sometimes with a combination of both drugs (9%).

Management of antithrombotics included assessment of plasmatic coagulation or platelet function, which influenced the timing of surgery: Provided that no urgent indication was present, surgery was delayed for a median of 3 [2-4] days post-admission with the antithrombotics being terminated and the corresponding patients (29%) being clinically observed on the normal ward. Moreover, hemostatic therapy was applied in 55% of all such patients before or during surgery. Thereby, PCC (62%), phytomenadione (7%; Konakion®, Roche Pharmaceuticals, Grenzach, Germany) or PCC, and phytomenadione (19%) and in rare cases, fresh frozen plasma (FFP; 3%), were used for anticoagulants and desmopressin (55%; Minirin®, Ferring Arzneimittel, Kiel, Germany), tranexamic acid (18%; Cyklokapron®, Pfizer, Berlin, Germany), a combination of both drugs (10%), or platelet transfusions (12%) for antiplatelets.

Hematoma evacuation was performed by burr hole drainage in 93% of cases, while the remaining patients received a craniotomy. One or more subdural drains were placed in all patients. During the hospital stay, nine patients (1%) died from inter alia cardiopulmonary insufficiency, multiorgan dysfunction due to sepsis, or acute secondary hemorrhage with trans tentorial herniation. On discharge, 94% of the remaining patients had a GCS score between 13 and 15, and the median GOS and mRS scores were 5 [4-5] and 1 [1-3], respectively. While half of the patients needed secondary hospital or rehabilitation care, the other half could be discharged home. Within 30 days of the primary surgical intervention, the overall reoperation rate due to remaining or recurrent cSDH was 23%.

### Reoperation after cSDH recurrence

Reoperation within 30 days of the primary hematoma evacuation was performed in 145 cSDH patients (23%) due to either neurological deterioration or missing improvements caused by residual or recurrent hematomas. A burr hole trepanation was performed in 58% of those cases, while 42% of patients underwent a craniotomy during the second operation.

In the univariate logistic analysis, only the presence of comorbidities in general, as well as arterial hypertension and renal insufficiency as comorbidities, were found to be significant predictors for the need for reoperation within 30 days (Table 1). Moreover, in the multivariate analysis that included age, gender, known comorbidities, antithrombotic medication, and cSDH thickness as covariates, only a history of chronic comorbidity (OR 2.12; 95% CI, 1.30–3.55; *p* = 0.003) was found to be an independent predictor for reoperation as well.

**Table 1:** Univariate and multivariate analyses of predictive factors for reoperation due to cSDH recurrence

Characteristics	No reoperation	Reoperation	Univariate analysis *p*-value	Multivariate analysis *p*-value	Multivariate analysis OR (95% CI)
No. of patients	478	145	-	-	-
Gender			0.581	0.504	1.18 (0.72–1.90)
Male	328 (69%)	103 (71%)			
Female	150 (31%)	42 (29%)			
Median age [IQR]	75 [68–82] years	75 [69–80] years	0.482	0.192	0.99 (0.97–1.00)
Antithrombotics	234 (44%)	84 (54%)	0.059	0.163	1.20 (0.81–1.77)
Anticoagulants	109 (23%)	42 (29%)	0.193		
Antiplatelets	117 (24%)	42 (29%)	0.384		
Both	8 (2%)	0 (0%)	0.076		
Comorbidities					
Known comorbidities	280 (59%)	111 (77%)	***p < 0.001***	**0.003**	**2.12 (1.30–3.55)**
Arterial hypertension	152 (32%)	63 (43%)	**0.010**		
Cardiac arrhythmias	90 (19%)	38 (26%)	0.055		
Coronary artery disease	53 (11%)	25 (17%)	0.052		
Stroke history	26 (5%)	9 (6%)	1.000		
Diabetes mellitus	63 (13%)	24 (17%)	0.306		
Malignancy	41 (9%)	14 (10%)	0.689		
Renal insufficiency	27 (6%)	16 (11%)	**0.028**		
Alcohol abuse	11 (2%)	4 (3%)	0.753		
Median laboratory values [IQR]					
INR	1.03 [0.87–1.09]	1.04 [0.88–1.14]	0.768		
aPTT (s)	25.1 [23.3–27.3]	24.9 [23.4–27.5]	0.981		
Platelet count (10^9^/L)	243 [194–303]	242 [193–321]	0.458		
Creatinine (mg/dL)	0.84 [0.73–1.02]	0.85 [0.70–1.07]	0.219		
GFR (mL/min/1.73m^2^)	81 [66–92]	79 [63–92]	0.568		
Initial clinical presentation					
GCS [IQR]	15 [14–15]	14 [14–15]	0.268		
mRS [IQR]	2 [2–3]	3 [2–3]	0.095		
Midline shift	330 (82%)	89 (79%)	0.595		
Median cSDH thickness [IQR]	20 [15–25] mm	22 [16–27] mm	0.238	0.177	1.02 (0.99–1.05)

*aPTT*, activated partial thromboplastin time; *GCS*, Glasgow Coma Scale; *GFR*, glomerular filtration rate; *INR*, international normalized ratio; *IQR*, inter quartile range; *mRS*, modified Rankin scale

### Patients with anticoagulants, antiplatelets, and without antithrombotics

In our study, 151 patients were on anticoagulant and 159 patients were on antiplatelet medication, respectively (Table 2). Compared to patients without antithrombotic medication, those patients were significantly older (*p* < 0.001 each) and predominately males (*p* = 0.0432 each; Supplement 1).

**Table 2:** Characteristics, clinical course, and outcome in cSDH patients with anticoagulant, antiplatelet, and no antithrombotic medication

Characteristics	No antithrombotics	Anticoagulation	Antiplalelet	*p*-value
No. of patients	305	151	159	-
Gender				**0.006**
Male	193 (63%)	114 (76%)	119 (75%)	
Female	112 (37%)	37 (24%)	40 (25%)	
Median age [IQR]	73 [63–79] years	77 [71–80] years	77 [70–84| years	** < 0.001**
Initial clinical presentation				
GCS 13–15	272 (89%)	129 (85%)	140 (88%)	0.428
GCS 9–12	17 (6%)	16 (11%)	13 (8%)	0.150
GCS < 9	13 (4%)	5 (3%)	5 (3%)	0.788
mRS [IQR]	2 [2, 3]	3 [2, 3]	3 [2, 3]	0.164
Comorbidities				
Known comorbidities	155 (51%)	125 (83%)	103 (65%)	** < 0.001**
Arterial hypertension	79 (26%)	66 (44%)	66 (42%)	** < 0.001**
Cardiac arrhythmias	21 (7%)	90 (60%)	11 (7%)	** < 0.001**
Coronary heart disease	18 (6%)	26 (17%)	31 (19%)	** < 0.001**
Stroke history	6 (2%)	13 (9%)	15 (9%)	** < 0.001**
Diabetes mellitus	25 (8%)	26 (17%)	35 (16%)	** < 0.001**
Renal insufficiency	10 (3%)	17 (11%)	15 (9%)	**0.002**
Alcohol abuse	11 (4%)	3 (2%)	1 (1%)	0.131
Complications				
Complications: cardiovascular	4 (1%)	10 (7%)	3 (2%)	**0.003**
Complications: pulmonary	11 (4%)	8 (5%)	12 (8%)	0.181
Complications: coagulation	5 (2%)	8 (5%)	0 (0%)	**0.004**
Complications: neurological	46 (15%)	35 (23%)	39 (25%)	**0.022**
Outcome				
In-hospital mortality	5 (2%)	1 (1%)	3 (2%)	0.628
GOS at discharge [IQR]	5 [5–5]	5 [4–5]	5 [4–5]	0.595
mRS at discharge [IQR]	1 [1–3]	2 [1–3]	2 [1–3]	0.262
Reoperation < 30 days	61 (20%)	42 (28%)	42 (26%)	0.112

*aPTT*, activated partial thromboplastin time; *GCS*, Glasgow Coma Scale; *GFR*, glomerular filtration rate; *GOS*, Glasgow Outcome Scale; *INR*, international normalized ratio; *mRS*, modified Rankin scale

A mild brain injury (GCS 13–15) was documented in 85% and 88% of patients on anticoagulants and antiplatelets, respectively, vs. in 89% of patients without antithrombotics. A moderate injury (GCS 9–12) was present in 11% (anticoagulant) and 8% (antiplatelet) vs. 6% (no antithrombotic medication) and a severe injury (GCS < 9) in 3% (anticoagulant) and 3% (antiplatelet) vs. 4% (no antithrombotic medication).

Known comorbidities were most frequently found in patients on anticoagulants (83%), and thus significantly more common than in patients on antiplatelets (65%; *p* = 0.001; Supplement 1). Compared to patients without antithrombotic medication, however, known comorbidities (51%) were found significantly more often in both, the anticoagulants (*p* < 0.001) and the antiplatelets (*p* = 0.010) groups. As expected, cardiac arrhythmias were predominantly found in patients on anticoagulants (60%), while patients on antiplatelets most often suffered from arterial hypertension (42%). Furthermore, anticoagulant and antiplatelet medication were both significantly associated with the comorbidities stroke (*p* = 0.005 and *p* = 0.003), diabetes mellitus (*p* = 0.018 and *p* < 0.001), and renal insufficiency (*p* = 0.008 and *p* = 0.028). Significant inter-group differences could also be observed in the occurrence of cardiovascular and coagulative complications, which were most frequently found in patients on anticoagulants (7% and 5%, respectively), while neurological complications were highest in the antiplatelets group (25%; Table 2). A significantly higher burden of cardiovascular complications was documented in patients on anticoagulants compared to patients without antithrombotic medication (*p* = 0.028; Supplement 1).

There were, however, no significant differences regarding in-hospital mortality, GOS, mRS, or reoperation risk within 30 days between patients on anticoagulants, on antiplatelets, or without antithrombotic medication.

## Discussion

### Clinical management of patients with antithrombotics

The incidence rate of chronic subdural hematomas is rising and has been associated with the increasing use of antithrombotics [17, 28]. Patients with cSDH will, therefore very frequently present with antithrombotic medication and chronic comorbidities that need to be considered in their clinical management.

In contrast to other reported strategies, where patients on antiplatelet drugs with cSDH routinely receive platelet transfusions, only a few such patients received a platelet transfusion before surgery in this study. In recent years, the use of platelet transfusions in emergencies such as intracranial bleedings has been questioned due to reports of lacking benefit or even detrimental effects [2, 9, 20]. In the prospective, multi-center PATCH-study, platelet transfusion was concluded to be inferior to standard care in patients with spontaneous intracerebral hemorrhage. It was associated with higher complication and mortality rates [5]. Our study suggests that cSDH patients on antiplatelet drugs can have an excellent clinical outcome, as measured by GOS, without the extensive use of platelet transfusions. Instead, assessing platelet function and a short delay of the surgical intervention under clinical observation or hemostatic treatment with, e.g., desmopressin or tranexamic acid in more urgent cases seem to be sufficiently effective management strategies. Of note, the addition of tranexamic acid to standard surgical cSDH drainage has been associated with a delay of hematoma recurrence and a reduction of residual hematoma volume in a recent prospective randomized trial, indicating that it might be the preferrable choice when hemostatic management of cSDH patients on antiplatelets is required [45].

Anticoagulants such as VKA have traditionally been reversed with FFP. However, delay in hemostasis correction and potential volume overload make FFP not an ideal reversal agent [23]. A rapid hemostasis correction without volume overload can be achieved with PCC. While this compound has been implemented in European guidelines for rapid anticoagulation reversal and has been routinely used in European countries for many years, 4-factor PCC, as used in this study, was not approved in the USA until 2013. In our study, most patients with cSDH and anticoagulant medication received PCC prior to surgery (62%). Of note, the rate of complications related to coagulation was significantly increased in patients on anticoagulants in general, underlining that caution might still be warranted when reversing anticoagulation.

Surprisingly, a considerable number of patients (15%) had not received any reversal of their anticoagulants prior to surgery. We attribute this to the fact that reversal of direct Xa-inhibitors with the corresponding drugs or with PCC has been proven to be feasible and effective only recently and had therefore not been performed in this retrospective analysis between 2006 and 2016. Our current management of such cSDH patients, however, includes, depending on the severity of symptoms, admission for observation, and monitoring of drug activity until surgery can be safely performed as well as the urgent reversal of the direct Xa-inhibitors with PCC or specific antagonists if necessary.

### History of chronic disease as an independent predictor for reoperation

**Fig. 2:**
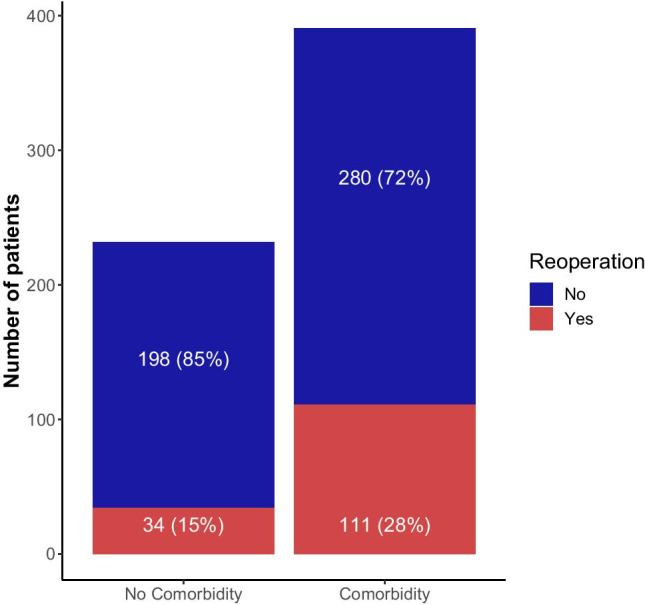
The proportion of patients requiring cSDH reoperation was significantly higher in the patient group with known comorbidities

Recurrence of the hematoma requiring surgery is a frequent complication in patients with cSDH. More than 20 factors have been suggested as predictors for cSDH recurrence in several studies, reflecting the difficulty in classifying and predicting this disease’s course [7, 33, 34, 42, 44]. Especially antithrombotics, and more specifically Clopidogrel and Warfarin, are highly debated as risk factors for recurrences [33, 38]. Several authors report results both in favor and against this notion, and final conclusions cannot be drawn [3, 15, 33–35, 39, 44]. In this study, we found no link between antithrombotics and an increased reoperation risk due to residual or recurrent hematomas within 30 days after surgery in the univariate or multivariate analysis. Moreover, despite our data showing that a larger proportion of patients with either anticoagulants or antiplatelets underwent a reoperation than patients with no antithrombotics, these differences did not reach statistical significance. Instead, the prevalence of chronic diseases in the patient history was identified as an independent predictor for reoperation (Fig. 2). Therefore, the fact that patients under antithrombotics had more rebleeding might not be explained by the antithrombotics themselves but by the presence of the underlying chronic diseases.

**Fig. 3:**
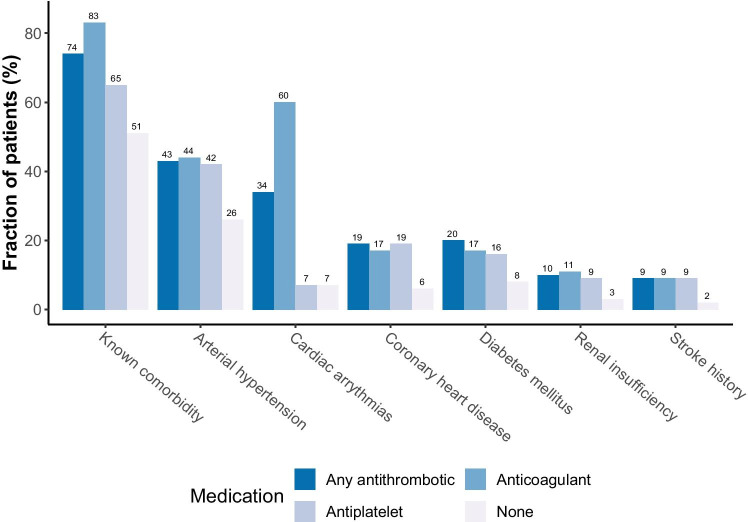
Patients on antithrombotics were more commonly presenting with chronic comorbidities than patients without antithrombotics

Group comparisons between the patients with anticoagulant vs. antiplatelet vs. no antithrombotic medication showed several distinctive and significant differences: Patients on either anticoagulants or antiplatelets were generally older and presented more commonly with chronic comorbidities (Fig. 3). Systemic, chronic diseases are known to predispose to vascular complications and increased bleeding tendency. Arterial hypertension, for example, is a known risk factor for post-craniotomy intracranial hemorrhage, and diabetes mellitus is reported to increase the risk for intracranial hemorrhages [8, 11]. An increased bleeding tendency due to, among others, platelet dysfunction has also been shown in patients with renal insufficiency, and an association has been made between coronary heart disease and traumatic intracranial hemorrhages in a large, population-based study, with atherosclerotic vessel changes as a likely shared underlying pathology [25, 27]. In our study, the prevalence of arterial hypertension and renal insufficiency was significantly higher in the patient group with revision surgery. Results similar to ours have been reported in several other studies, with chronic diseases such as hypertension and diabetes mellitus being independent risk factors for hematoma recurrence [37, 42, 44]. A history of stroke has also been identified as an independent predictor for cSHD recurrence in a study by Okano et. al [36].

It is, therefore, important for clinicians to consider chronic comorbidities in patients when evaluating the risk for hematoma recurrence. Moreover, given the increased interest in minimally invasive procedures for the treatment of cSDH, such as embolization of the middle meningeal artery, which claim to reduce the recurrence rates compared to cSDH surgery, an emphasis on chronic comorbidities, as proposed by our data, might be relevant for the design of future, prospective studies [18].

While not the focus of the current study, the association of peri-operative steroid use (which was not part of our treatment regimen for cSDH) with the necessity of repeat operations has been the focus of recent investigations and is currently under critical discussion: Several reports on beneficial effects of steroids on cSDH recurrence exist, but some authors also observed contradictory and even adverse effects [12, 19, 24, 48]. Correspondingly, the very recently published multicenter, randomized DEX-CSDH trial has reported fewer repeat operations but at the same time also fewer favorable outcomes and more adverse events with a 2-week oral dexamethasone treatment compared to placebo at 6 months [21]. Although more prospective studies are currently underway, especially in more vulnerable patients with chronic comorbidities, the peri-operative administration of steroids should thus be evaluated with caution [29, 30].

### Clinical outcome is not worsened by the use of antithrombotics

While a vast number of authors have tried to investigate the association between antithrombotics and hematoma recurrence rates in cSDH patients, only a few studies have examined the impact of antithrombotics on the clinical outcome itself. Those few studies have shown very different results, ranging from anticoagulants as negative predictors for good outcome, neither anticoagulants nor antiplatelets as predictive factors for poor outcome, to antiplatelets as even a predictor for a good outcome [1, 22, 41].

This study examined the clinical outcome (GOS, mRS) of 151 cSDH patients on anticoagulants and 159 cSDH patients on antiplatelets after surgical hematoma removal and compared findings to a cohort of cSDH patients without such antithrombotic medication. The in-hospital mortality rate was 1% for anticoagulants, 2% for antiplatelets, and 2% for no-antithrombotics (no significant difference). In our study, antithrombotics were thus not linked to higher mortality in cSDH patients, which is in line with similar findings by Fornebo et al. [15]. The median GOS at discharge was 5 in all groups. However, the median mRS was lower in the no-antithrombotics group (1) compared to the anticoagulants and antiplatelets group (both 2), without reaching a significant difference. Similarly, in the multicenter prospective cohort study by Poon et al., neither antiplatelet nor anticoagulant drug use was associated with worse clinical outcomes after cSDH surgery [39]. However, in a recent study by Atsumi et al. [4], comorbidities such as hemodialysis and chronic heart failure were linked to the impairment of activities of daily living in patients with cSDH, while similar to our findings, antithrombotics themselves were no independent risk factor. Taken together, these data may indicate that neither anticoagulants nor antiplatelets necessarily lead to a worse clinical outcome in surgically treated cSDH patients if managed adequately.

Our findings, therefore, may support the notion that surgical intervention for cSDH is highly indicated even in patients on antithrombotics, as their chances for a good clinical outcome (GOS 5) can be considered very good.

## Limitations

Further studies are needed to determine optimal protocols for the reversal of anticoagulants and antiplatelets and, more importantly, for the clinical management of patients with chronic comorbidities and a high risk for recurrent bleedings, as this study comes with several limitations: Due to its retrospective design, it might be subject to selection and information biases albeit its large cohort of 623 patients. While the surgical, as well as the clinical management of cSDH patients, has remained unchanged during the 10-year course of the study, including, e.g., the strict peri-operative avoidance of steroids, management of antithrombotics might have varied. A prospective study is needed to minimize those biases. Although we aimed to exclude acute on chronic subdural hematomas in this analysis, the lack of a uniform definition of this entity might be a further bias, limiting the generalization of the results [10]. Also, we did not further investigate the association of each type of antithrombotic agent (e.g., DOAKs) to reoperation, as subgroups were too small for statistical analysis. Another limitation might be our clinical practice to limit postoperative CT scans to patients without improvement of preoperative symptoms or neurological deterioration, which, in the absence of routine follow-up CT scans, makes it difficult to differentiate between residual or recurrent bleedings in cases of revision surgery. Recent data has, however, supported this practice, showing no impact on patient management of routine follow-up CT [16] and even suggesting a correlation with increased rate of revision surgeries [43]. Finally, limitations due to the assessment of cSDH reoperations within only 30 days after the primary surgery have to be noted. Potential hematoma recurrences at later timepoints were thus not considered and additional studies with longer follow-up periods are warranted.

## Conclusion

**Fig. 4:**
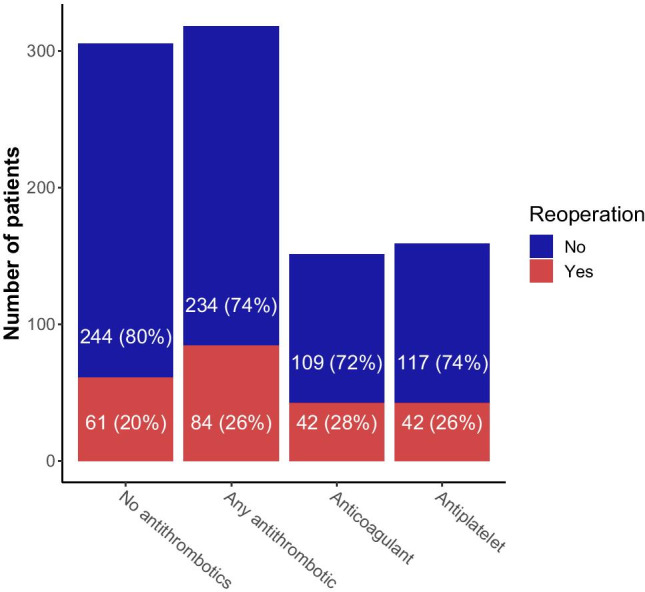
Patients on antithrombotics did not have a significantly higher risk of reoperation in univariate and multivariate analysis

In conclusion, our current data show that patients on antithrombotics have no significantly higher risk of reoperation due to residual hematomas or rebleeding compared to patients without antithrombotics in multivariate analysis (Fig. 4). However, a history of chronic diseases was identified as an independent predictive factor for the need for reoperation within 30 days after initial hematoma evacuation. An increased recurrence rate in patients on antithrombotics might thus not be explained by the medication itself but by the presence of chronic comorbidities such as hypertension and renal insufficiency, which are distinctively more common in such patients and might predispose to rebleeding. Neither the mortality rate nor the clinical outcome was worse in patients on anticoagulants or antiplatelets after evacuation surgery than patients without antithrombotics.

## Supplementary Information

Below is the link to the electronic supplementary material.

Supplementary file1 (DOCX 17.2 KB)

## Data Availability

The datasets analyzed during the current study are available from the corresponding author on reasonable request.
