# Supplementary material for: Relevance of comorbidities and antithrombotic medication as risk factors for reoperation in patients with chronic subdural hematoma
Source: Neurosurg Rev. 2021 Jul 9;45(1):729–39. doi: 10.1007/s10143-021-01537-x (PMC8827308; doi:10.1007/s10143-021-01537-x)
Supplement: Supplementary file 1 — Supplementary file1 (DOCX 17.2 KB) [file 10143_2021_1537_MOESM1_ESM.docx]

**Supplement 1: Post-hoc analysis**

|  | **Comparison** | **Adjusted p-value** |
| --- | --- | --- |
| **Gender** | Anticoagulation vs. No Antithrombotics  Anticoagulation vs. Antiplatelet  No Antithrombotics vs. Antiplatelet | 0.0432  1.0000  0.0432 |
| **Age** | Anticoagulation vs. No Antithrombotics  Anticoagulation vs. Antiplatelet  No Antithrombotics vs. Antiplatelet | <0.001  0.971  <0.001 |
| **GCS 13-15** | Anticoagulation vs. No Antithrombotics  Anticoagulation vs. Antiplatelet  No Antithrombotics vs. Antiplatelet | 1  1  1 |
| **GCS 9-12** | Anticoagulation vs. No Antithrombotics  Anticoagulation vs. Antiplatelet  No Antithrombotics vs. Antiplatelet | 0.444  1  1 |
| **GCS <9** | Anticoagulation vs. No Antithrombotics  Anticoagulation vs. Antiplatelet  No Antithrombotics vs. Antiplatelet | 1  1  1 |
| **mRS at presentation** | Anticoagulation vs. No Antithrombotics  Anticoagulation vs. Antiplatelet  No Antithrombotics vs. Antiplatelet | 0.252  0.713  0.367 |
| **Known comorbidities** | Anticoagulation vs. No Antithrombotics  Anticoagulation vs. Antiplatelet  No Antithrombotics vs. Antiplatelet | <0.001  0.001  0.010 |
| **Arterial**  **hypertension** | Anticoagulation vs. No Antithrombotics  Anticoagulation vs. Antiplatelet  No Antithrombotics vs. Antiplatelet | 0.001  1  0.002 |
| **Cardiac arrythmias** | Anticoagulation vs. No Antithrombotics  Anticoagulation vs. Antiplatelet  No Antithrombotics vs. Antiplatelet | <0.001  <0.001  1 |
| **Coronary heart disease** | Anticoagulation vs. No Antithrombotics  Anticoagulation vs. Antiplatelet  No Antithrombotics vs. Antiplatelet | <0.001  1  <0.001 |
| **Stroke** | Anticoagulation vs. No Antithrombotics  Anticoagulation vs. Antiplatelet  No Antithrombotics vs. Antiplatelet | 0.005  1  0.003 |
| **Diabetes mellitus** | Anticoagulation vs. No Antithrombotics  Anticoagulation vs. Antiplatelet  No Antithrombotics vs. Antiplatelet | 0.018  0.656  <0.001 |
| **Renal insufficiency** | Anticoagulation vs. No Antithrombotics  Anticoagulation vs. Antiplatelet  No Antithrombotics vs. Antiplatelet | 0.008  1  0.028 |
| **Alcohol abuse** | Anticoagulation vs. No Antithrombotics  Anticoagulation vs. Antiplatelet  No Antithrombotics vs. Antiplatelet | 1  1  1 |
| **Cardiovasc. complications** | Anticoagulation vs. No Antithrombotics  Anticoagulation vs. Antiplatelet  No Antithrombotics vs. Antiplatelet | 0.0276  0.1990  1.000 |
| **Pulmonary complications** | Anticoagulation vs. No Antithrombotics  Anticoagulation vs. Antiplatelet  No Antithrombotics vs. Antiplatelet | 1  1  0.566 |
| **Coagulative complications** | Anticoagulation vs. No Antithrombotics  Anticoagulation vs. Antiplatelet  No Antithrombotics vs. Antiplatelet | 0.154  0.054  0.458 |
| **Neurological**  **complications** | Anticoagulation vs. No Antithrombotics  Anticoagulation vs. Antiplatelet  No Antithrombotics vs. Antiplatelet | 0.125  1  0.098 |
| **In-hospital**  **mortality** | Anticoagulation vs. No Antithrombotics  Anticoagulation vs. Antiplatelet  No Antithrombotics vs. Antiplatelet | 1  1  1 |
| **GOS at discharge** | Anticoagulation vs. No Antithrombotics  Anticoagulation vs. Antiplatelet  No Antithrombotics vs. Antiplatelet | 0.990  0.744  1.000 |
| **mRS at discharge** | Anticoagulation vs. No Antithrombotics  Anticoagulation vs. Antiplatelet  No Antithrombotics vs. Antiplatelet | 0.358  0.612  0.635 |
| **Reoperation <30 days** | Anticoagulation vs. No Antithrombotics  Anticoagulation vs. Antiplatelet  No Antithrombotics vs. Antiplatelet | 0.396  1  0.396 |

GCS: Glasgow Coma Scale; GOS: Glasgow Outcome Scale; mRS: modified Rankin scale
